# The Impact of Dietary Fiber on Cardiovascular Diseases: A Scoping Review

**DOI:** 10.3390/nu17030444

**Published:** 2025-01-25

**Authors:** Lu Zhang, Yifei Chen, Qiaoren Yang, Jun Guo, Siyu Zhou, Tian Zhong, Ying Xiao, Xi Yu, Ke Feng, Ye Peng, Zhong Han, Feifei Feng, Ling Wang

**Affiliations:** 1 Faculty of Medicine, Macau University of Science and Technology, Macau 999078, China; 3230004406@student.must.edu.mo (L.Z.); 2230017811@student.must.edu.mo (Y.C.); 2230004813@student.must.edu.mo (Q.Y.); 2230004841@student.must.edu.mo (S.Z.); tzhong@must.edu.mo (T.Z.); yxiao@must.edu.mo (Y.X.); xyu@must.edu.mo (X.Y.); kfeng@must.edu.mo (K.F.); pengye@must.edu.mo (Y.P.); 2 Department of Nursing, Zunyi Medical University, Zhuhai Campus, Zhuhai 519041, China; guojun@zmuzh.edu.cn; 3 School of Food Science and Engineering, South China University of Technology, Guangzhou 510641, China; fezhonghan@scut.edu.cn; 4 College of Public Health, Zhengzhou University, Zhengzhou 450002, China; feifeifeng@zzu.edu.cn

**Keywords:** cardiovascular disease, dietary fiber, scoping review, health promotion

## Abstract

**Background/Objectives**: Cardiovascular disease (CVD) remains a leading cause of morbidity and mortality globally, placing an ever-increasing burden on healthcare systems. Dietary factors play a crucial role in the development and progression of CVD. Among them, dietary fiber has emerged as a potential modifiable factor with the potential to impact CVD risk. However, the specific and independent effects of dietary fiber on CVD are still not fully understood, making this area of research both challenging and of great significance. **Methods**: The publications of human studies involving the impact of dietary fiber on CVD were retrieved from databases including PubMed, Embase, Web of Science, Scopus, Cochrane Library, CBM, and China National Knowledge Infrastructure (CNKI). A search was conducted within these databases for studies published between 2014 and 20 March 2024. The included literature was screened and summarized. **Results**: A total of seven articles were included, and the related studies encompassed various types of dietary fiber, including soluble and insoluble dietary fiber, as well as research from different countries and regions. The outcome indicators involved an important measure known as the hazard ratio (HR). **Conclusions**: Increasing the intake of dietary fiber could reduce the risk of cardiovascular diseases through various mechanisms. To increase the consumption of dietary fiber from multiple sources, it would be beneficial to develop and promote healthcare interventions to enhance people’s awareness of the health benefits of dietary fiber, promote the consumption of fiber-rich foods, and advocate for a healthier diet.

## 1. Introduction

Non-communicable diseases represent a significant public health issue and remain the leading cause of death globally; of these, cardiovascular diseases (CVDs) account for the majority of cases and pose a major threat to human health [1,2,3,4,5,6,7], with a high mortality rate, especially among individuals of middle age and above. Researchers have conducted systematic and in-depth investigations into the prevention and treatment of these diseases [8,9,10,11,12]. An unhealthy diet is one of the modifiable risk factors for CVD. Dietary fiber includes soluble fiber and insoluble fiber, which are components of plant-source food. It cannot be digested by the enzymes of the gastric–intestinal duct in humans but plays important roles in human health.

CVD is a broad term, encompassing a group of diseases involving the heart and blood vessels, including coronary heart disease (CHD), hypertension, stroke, and more. One of the most effective methods to reduce the risk of developing CVD is altering one’s diet to decrease the blood level of low-density-lipoprotein cholesterol; dietary fiber might help with this. It has been reported that insufficient intake of dietary fiber is closely associated with various health risks [13,14,15,16,17]. Dietary fiber can be divided into soluble and insoluble types. Soluble dietary fiber could come from grains, legumes, nuts, fruits, and vegetables. Increasing the intake of soluble dietary fiber has attracted great attention, as it can help to reduce the risk of diseases. Soluble dietary fiber can dissolve in water, forming a gel-like substance which can adsorb bile salt in the small intestine and then increase the discharge of bile from the gut; in this way, soluble dietary fiber helps to decrease blood cholesterol. By the same mechanism, soluble fiber adsorbs simple sugars digested in the gut and prevents the sugars from being absorbed by the small intestine, thus reducing postprandial blood glucose. Insoluble dietary fiber cannot dissolve in water but can expand fecal bulk through holding water and then help gut movement, which is a way to prevent constipation, especially in the elderly. Recently, substantial studies have demonstrated that indigestible fiber in the large intestine can be fermented and broken down by the intestinal flora and then produce short-chain fatty acids and other chemicals which are beneficial to human health [18,19].

## 2. Materials and Methods

### 2.1. Search Strategy and Selection

Computer retrieval was the main method used in this study, with manual retrieval used as a supplement. The databases used for retrieval included PubMed, Web of Science, Embase, CBM, Cochrane, and China National Knowledge Infrastructure (CNKI), and the reference lists of the included studies were also tracked. There were no restrictions on the publication type for the literature retrieval, and journal articles, dissertations, and conference papers were all included. A combination of subject terms and free words was used for retrieval, with searches conducted in Chinese or English, depending on the characteristics of each database. Corresponding search strategies were developed accordingly. Secondary searches were performed on the references of the included studies and relevant reviews.

During the initial screening of the retrieved articles, a series of exclusion criteria were applied. Methodological quality was formally assessed by using a pre-defined set of criteria. For studies, we evaluated the study design. Only human-based studies were considered relevant; thus, non-human studies were excluded. Regarding study design, preference was given to well-controlled observational studies and randomized controlled trials. Studies with a small sample size that might not provide sufficient statistical power to detect the relationship between dietary fiber and CVD were excluded. Additionally, studies that failed to adequately control for confounding factors, such as other dietary components, lifestyle factors, and pre-existing medical conditions, were also removed from further consideration. Articles were also excluded if they were found to be irrelevant to the research question. For example, those focusing on dietary components other than dietary fiber in relation to CVD or studies that did not report any data related to the impact of dietary fiber on CVD were excluded.

In addition to the electronic database searches, a manual search was conducted to identify potentially relevant studies that might have been missed in the automated searches. The manual search was carried out by screening the reference lists of all included systematic reviews and meta-analyses identified in the initial database search. We also searched the reference lists of highly cited original research articles on the topic of dietary fiber and CVD. The time period for this manual search was parallel to the electronic search, covering the period from the establishment of the databases in 2014 to 20 March 2024. Specific keywords related to dietary fiber, CVD, and their interrelationships were used to guide the manual search.

The retrieval process was conducted in accordance with the guidance provided by the PRISMA extension for Scoping Reviews (PRISMA-ScR), which consists of four stages: searching, screening at the initial level, application of inclusion/exclusion criteria, and synthesis. In 2016, Tricco et al. [20] made adjustments to the core version of PRISMA to develop a checklist specifically designed for scoping review reports called the PRISMA extension for Scoping Reviews (PRISMA-ScR). This was published in the Annals of Internal Medicine in 2018. The development process was based on guidelines released by the Enhancing the Quality and Transparency Of health Research (EQUATOR) Network.

The search terms and search expressions were as follows: (Cardiovascular disease OR Cardiovascular OR Cardiometabolic Risk Factor OR Factor, Cardiometabolic Risk OR Risk Factor, Cardiometabolic OR Risk Factors, Cardiometabolic) AND (Diet fiber OR fiber OR Dietary Fibers OR Fibers, Dietary OR Fiber, Dietary OR Wheat Bran OR Bran, Wheat OR Brans, Wheat OR Wheat Brans OR Roughage OR Roughages).

### 2.2. Eligibility Criteria: Inclusion and Exclusion

The inclusion criteria were set to include relevant literature on the association between dietary fiber and CVD from 2014 to the present studies (excluding basic or animal experiments). The literature should primarily consist of observational studies, including cohort studies, case–control studies, and large-scale epidemiological surveys. Only primary research data were included rather than reviews. The study population should include large samples of individuals; for cross-sectional studies, representative populations with large sample sizes should be included; for cohort studies, individuals without CVD at the beginning of the study should be selected as subjects to avoid excessive heterogeneity in the study population. Finally, outcome measures should include relative risk (RR) or hazard ratio (HR), which reflect the risk of developing CVD in the populations exposed to dietary fiber.

Exclusion criteria were studies with small sample sizes, case reports, case-series reports, research that did not involve the correlation between dietary fiber and CVD risk, non-Chinese or English literature, duplicate publications, studies with incomplete data reporting that could not be retrieved, and studies that had only an abstract available and the full text inaccessible. Studies with unavailable statistical analysis data, unclear research type, or poorly designed experiments were excluded.

### 2.3. Data Extraction

A form for data extraction was established to describe the general characteristics of the selected studies, such as study type, sample size, follow-up time, and dietary factors. The effect indicators, such as RR or HR values, were extracted to describe the research results of the impact of dietary fiber on CVD.

## 3. Results

### 3.1. Literature Screening and Results

**Figure 1:**
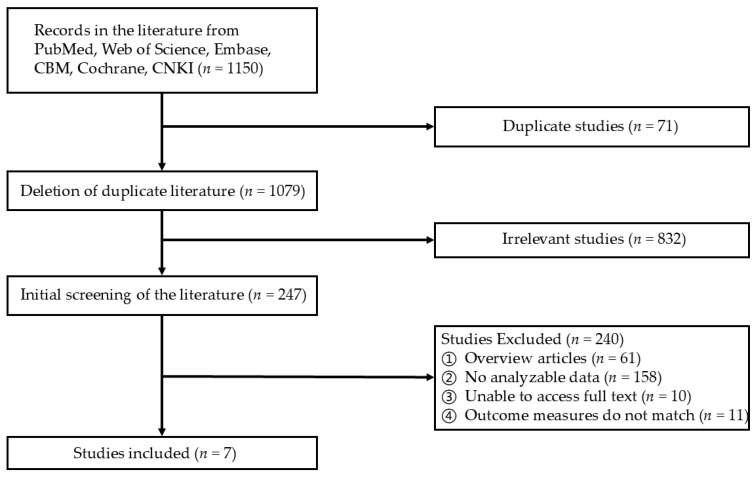
The flowchart of document selection.

By searching the database, a preliminary search within the designated time range yielded 1150 relevant articles. After removing the duplicate publications, 1079 articles remained. The titles and abstracts of these articles were then subjected to initial screening, resulting in the further elimination of 832 obviously irrelevant papers. Following guidelines provided beforehand to ensure methodological consistency, the full texts of potentially relevant articles (240 in total) were obtained. If the full texts could not be accessed, they were directly excluded from consideration. After completing the literature review process, a final selection of seven studies—all prospective cohort studies published in English—were included in the review (as shown in Figure 1).

### 3.2. Basic Characteristics of Included Studies

A total of 731,879 participants were included in the study across seven cohorts. The basic information of the included studies is shown in Table 1. The scope of this review encompasses various CVDs, including coronary heart disease (CHD) and aortic stenosis. The review consists of seven prospective studies conducted in various countries/regions, including Iran, the United States, Spain, France, China, the United Kingdom, and Malmö in Sweden. The review involved sample sizes ranging from 2,295 to 461,047 individuals, with the longest follow-up duration lasting up to 20 years. The age of the subjects ranged from 18 to over 70 years old, making the study scope quite extensive. The studies mainly focused on soluble dietary fibers, including grain fiber, legume fiber, nut fiber, vegetable fiber, and fruit fiber. The endpoint indicator was the hazard ratio (HR; see Table 2). All seven publications showed a significant decrease in CVD risk with the increase in dietary fiber intake.

### 3.3. Assessment Through HR

An illustration of these assessments is provided in Table 2. Some sources of soluble dietary fiber are negatively correlated with the risk of CVD. Among soluble dietary fibers, those from legumes, fruits, and vegetables are negatively associated with the risk of CVD. The dietary intake of grain and nut fiber does not show a significant correlation with the risk of CVD. In each cell of Table 2, the first value represents the hazard ratio (HR). The HR is a measure of the relative risk of CVD for the corresponding tertile/quintile (Q) of dietary fiber consumption, with the Q1 group serving as the reference (HR = 1). A value of HR > 1 indicates a higher risk of CVD compared with the reference group, while HR < 1 indicates a lower risk.

The second and third values in each cell represent the lower and upper bounds of the 95% confidence interval (95% CI) for the HR. The 95% CI reflects the precision of the HR estimate. If the 95% CI does not include 1, it suggests a statistically significant association between the dietary fiber consumption level of that group and the risk of CVD at the 5% significance level. A wider 95% CI implies less precision in the HR estimate, while a narrower CI indicates more confidence in the value. This information is presented to provide a comprehensive understanding of the relationship between dietary fiber intake and the risk of CVD among different studies.

The included studies had adjusted for various CVD risk factors and dietary variables by using different research methods. Ref. [21] adjusted for dietary intake of total fat (expressed as a percentage of daily energy), sodium (mg/1000 kcal), and vitamin C (mg/1000 kcal). In addition, according to the consumption of fruits (g/d) and vegetables (g/d) and extra virgin olive oil intake (g/d), adjustments were made in Ref. [22]. In the study [23], additional adjustments were made for a family history of CVD, previous angina or revascularization surgery, diabetes mellitus, hypertension, dyslipidemia, (hypercholesterolemia and/or hypertriglyceridemia), and depression. Ref. [25] adjusted the basic model according to age, gender, education level, daily energy intake, smoking status, alcohol consumption, and physical activity during leisure time. Ref. [26] adjusted for education level, income, smoking, alcohol consumption, BMI, overall physical activity, and a family history of diabetes or CVD, as well as intake of some types of food, e.g., fresh fruits, meat, and pickled vegetables. In the study [23], although an increase in fiber intake was not significantly associated with the incidence of CVD, an increase in whole-grain intake showed a negative correlation with the incidence of CVD.

**Table 2:** Hazard ratios (HRs) of CVD according to baseline tertiles/quintiles (Qs) of dietary fiber consumption (HR and 95 % CI).

References	Q1		Q2		Q3		Q4		Q5		P_trend_
	HR	95%CI	HR	95%CI	HR	95%CI	HR	95%CI	HR	95%CI	
Mirmiran P et al. [21]	1	-	0.67	0.35, 1.26	0.39	0.18, 0.83	-	-	-	-	0.050
Buil-Cosiales et al. [22]	1	-	0.92	0.67, 1.26	0.83	0.59, 1.16	0.71	0.50, 1.02	0.73	0.50, 1.06	0.083
Zazpe I. et al. [23]	1	-	0.95	0.60, 1.51	0.67	0.38, 1.16	-	-	-	-	0.130
Partula V.et al. [24]	1	-	0.91	0.76, 1.10	0.96	0.80, 1.15	0.94	0.78, 1.14	0.86	0.70, 1.06	0.190
Janzi S. et al. [25]	1	-	1.02	0.77, 1.36	0.84	0.62, 1.14	0.99	0.73, 1.35	0.85	0.60, 1.22	0.400
Yang J. et al. [26]	1	-	0.98	0.96, 1.00	0.97	0.94, 1.00	0.86	0.81, 0.93	-	-	0.002
Kelly R. et al. [27]	1	-	0.99	0.91, 1.09	0.92	0.84, 1.02	0.94	0.85, 1.05	-	-	-

The study in [21] from Iran suggested that higher intake of total, soluble, and insoluble dietary fibers was significantly associated with a reduced risk of CVD. The research study highlighted that an approximate 7% reduction in CVD risk was seen for every 10 g fiber intake. In the study [22], the PREDIMED trial findings indicated that an increased intake of dietary fiber, particularly from fruits, vegetables, or whole grains, was linked to a lower risk of CVD. The SUN Project’s study [23] associated a high Dietary Carbohydrate Index (CQI), indicative of quality carbohydrate intake (such as whole grains), with a lower risk of CVD. The NutriNet-Santé cohort study [24] found a significant association between higher dietary fiber intake and a decreased risk of CVD, various types of cancers, type 2 diabetes, and overall mortality. The comprehensive study [25] reinforced the role of fiber in overall health and longevity. Contrary to other findings, this study did not find a significant association between dietary fiber intake and the risk of aortic stenosis, a specific CVD condition. The research study in [26] from the China Kadoorie Biobank showed that higher consumption of coarse grains was associated with a lower risk of cardiometabolic diseases. The study from the UK Biobank [27] found that high intake of free sugars was associated with an increased risk of CVD, whereas high fiber intake was associated with a reduced risk. It emphasized the importance of fiber quality and the need to limit free sugars in the diet.

Across these studies, there is a consistent theme that higher intake of dietary fiber, especially from sources of fruits, vegetables, and whole grains, is beneficial for cardiovascular health. Insoluble fiber, which is mainly found in whole grains and vegetables, helps with intestinal health and may also contribute to heart health through mechanisms such as good control of glycemia.

## 4. Discussion

### 4.1. The Impact of Different Types of Dietary Fiber on CVD

Whole-grain flour, wheat bran, nuts, beans, and vegetables, such as cauliflower, green beans, and potatoes, are good sources of insoluble fiber. Soluble fiber has been shown to be able to reduce low-density-lipoprotein cholesterol (LDL-C). By binding with cholesterol particles in the digestive system, soluble fiber helps to decrease their absorption into the bloodstream. A diet high in fiber could also contribute to the maintenance of normal blood pressure levels. Some dietary fibers could reduce inflammation, a risk factor for coronary heart disease. High-fiber diets also help with weight management, which is crucial for heart health. There were different associations between CVD and individual foods or food groups, such as fruits and vegetables. The mechanisms behind the protective effects of fruits and vegetables against CVD are still unclear, but there are some biological foundations that could explain this association. By delaying the digestion and absorption of carbohydrates, dietary fiber could prevent postprandial hyperglycemia, enhance satiety, and lead to weight loss. The consumption of dietary fiber has been reported to be associated with a reduced risk of mortality, although the impact may vary depending on the type and quantity of fiber consumed.

Evidence regarding the effects of soluble versus insoluble fiber on mortality risk remains limited and inconsistent. The seven studies provided a comprehensive look at the role of dietary fiber in cardiovascular health. The Tehran lipid and glucose study by Mirmiran et al. [21] suggested that dietary fiber, including soluble and insoluble, is inversely associated with CVD risk. This is echoed in the PREDIMED trial by Buil-Cosiales et al. [22], who found that higher fiber intake, particularly from fruits, vegetables, and whole grains, was linked to reduced CVD risk. Zazpe et al. [23] from the SUN Project further supported these findings by associating high-quality carbohydrate intake, characterized by a higher proportion of whole grains, with lower CVD risk. The NutriNet-Santé cohort study by Partula et al. [24] expanded on this by showing that dietary fiber intake was inversely related not only to CVD but also to cancers, type 2 diabetes, and overall mortality. Janzi et al. [25] provided a more nuanced view, indicating no significant association between fiber intake and the risk of aortic stenosis, a specific cardiovascular condition. This highlights that the impact of dietary fiber may vary depending on the type of CVD. Yang et al. [26] demonstrated that coarse-grain consumption, a source of dietary fiber, was associated with a lower risk of cardiometabolic diseases in the Chinese population. Lastly, by comparing different types and sources of carbohydrates, the UK Biobank study by Kelly et al. [27] found that high intake of free sugars could increase CVD risk, while high fiber intake decreased it.

Based on the insights gleaned from the seven studies, we can formulate practical recommendations for reducing the risk of CVD through dietary fiber intake. These recommendations aim to integrate the findings into acceptable advice for individuals seeking to improve their heart health. The studies somewhat underscore the importance of consuming a variety of fiber sources. Soluble fibers, found in oats, legumes, apples, and citrus fruits, can help lower LDL-C and regulate blood sugar levels. Insoluble fiber present in whole grains, nuts, and vegetables like cauliflower and potatoes promotes intestinal health and may aid in weight management. Mixing both types of fiber ensures a broader range of benefits. Whole grains are a common theme across the studies for their positive impact on heart health. Replacing refined grains with whole grains contributes to a lower risk of CVD. Whole grains provide not only dietary fiber but also essential nutrients that support overall health. High-quality fiber, such as that found in whole grains, fruits, and vegetables, is associated with a lower risk of CVD. The quality of carbohydrates is as important as the quantity, with a focus on foods that have a low glycemic index and are rich in fiber. The UK Biobank study highlighted the detrimental effects of high free sugar intake on heart health. Reducing the consumption of sugary beverages, snacks, and processed foods could lower the risk of CVD. Instead, opt for naturally sweet foods like fruits, which provide the added health benefit of dietary fiber. The PREDIMED trial’s findings support the Mediterranean diet as a heart-healthy eating pattern. This diet is rich in dietary fiber from fruits, vegetables, legumes, nuts, seeds, and whole grains and also includes healthy fats from olive oil and fish. While increasing fiber intake is beneficial for health, it is also important to be mindful of portion sizes to maintain a healthy weight. Excess calories, even from healthy foods, could lead to weight gain, which is a risk factor for CVD. Fiber absorbs water, so adequate hydration is necessary to prevent constipation and support the digestive system. A sudden increase in fiber intake can lead to digestive discomfort. It is advisable to gradually increase fiber intake to allow the body to adjust and to ensure a sustainable dietary change. Individual responses to different types of fiber can vary. It is important to identify which fiber sources work best for the individual and to adjust one’s diet accordingly. Before making significant dietary changes, especially for those with existing health conditions, it is crucial to consult with healthcare providers. They can provide personalized advice and ensure that dietary changes are safe and appropriate. The collective evidence from these studies suggests that a diet rich in dietary fiber, particularly from whole grains, fruits, and vegetables, is beneficial for cardiovascular health. By focusing on high-quality, fiber-rich foods and adopting a Mediterranean diet pattern, individuals could significantly reduce their risk of CVD. These dietary changes, combined with regular physical activity and other healthy lifestyle choices, could lead to improved heart health and overall well-being. What should be kept in mind is that these recommendations should be tailored to individual needs and medical advice should be sought when necessary. However, the studies also suggested that not all fibers are equal in their impact on different cardiovascular conditions, and the overall dietary pattern plays a crucial role. For instance, the Mediterranean diet, rich in high-quality fibers, has been proven beneficial for heart health. All in all, these studies collectively affirmed the protective role of dietary fiber against CVD.

### 4.2. Challenges in Isolating the Impact of Dietary Fiber

One of the major challenges in this area of research is isolating the effect of dietary fiber from the complex matrix of nutrients present in fiber-rich foods. All foods identified as good sources of dietary fiber also contain numerous other bioactive components, many of which have been independently shown to confer benefits for the cardiovascular system. For example, fruits and vegetables, which are rich in dietary fiber, also contain antioxidants, vitamins, and minerals, which can contribute to cardiovascular health. As a result, disentangling the specific role of dietary fiber from the combined effects of these components is complex. Future studies should focus on innovative research designs, such as well-controlled intervention trials with purified fiber supplements or carefully designed dietary regimens that can isolate the effects of fiber. Additionally, advanced statistical methods may be employed to account for the confounding effects of other nutrients. In the current study, although we attempted to analyze the impact of dietary fiber on CVD, we acknowledge that the influence of these co-existing components cannot be completely ignored. This limitation should be considered when interpreting the results, and it highlights the need for further research to more precisely define the role of dietary fiber in preventing and managing CVD.

### 4.3. Insect-Derived Dietary Fibers: An Emerging Aspect

In addition to the traditional sources of dietary fiber, it is essential to consider the emerging role of fiber in insects and insect-derived foods. Insects are a staple food in many cultures, and their consumption is projected to increase globally due to their environmental sustainability, high protein content, and their potential as a rich source of various nutrients, including dietary fiber. Research indicates that insects contain different types of dietary fibers. For instance, chitin, a polysaccharide, is a major component of the insects’ exoskeleton and is classified as an insoluble fiber. Chitin has been associated with beneficial physiological effects, such as the modulation of gut microbiota and potential cholesterol-lowering properties, which are relevant to cardiovascular health. However, the exact impact of chitin and other insect-derived dietary fibers on CVD remains relatively understudied compared with traditional fiber sources. Limited studies have explored the overall cardiovascular effects of incorporating insect-based foods into the diet. Some suggest that the unique combination of nutrients in insects, including dietary fiber, might contribute to improved cardiovascular risk profiles. However, more research is needed to isolate the specific role of dietary fibers from insect-derived foods in influencing CVD risk factors, such as blood lipid levels, blood pressure, and endothelial function.

Future research could focus on rigorously designed intervention studies to evaluate the impact of consuming insect-based foods on CVD risk markers. These studies could also determine the optimal intake levels of insect-derived dietary fibers and assess their long-term effects on cardiovascular health. Understanding the role of insect-derived dietary fibers in CVD prevention and management could potentially lead to the development of novel dietary strategies and contribute to a more diverse and sustainable approach to cardiovascular health promotion.

### 4.4. Impact of Heterogeneity Among Studies

The seven studies included in this review display heterogeneity in terms of the populations and methods. This heterogeneity may have substantial implications for the results. Differences in population characteristics can significantly affect the associations between dietary fiber and CVD. For instance, variations in age can impact the metabolism of dietary fiber and the prevalence of CVD risk factors. Older populations may have distinct dietary patterns and a higher baseline risk of CVD, potentially modifying the observed effects of dietary fiber. Geographical location can also play a role, as different regions may have unique dietary habits, environmental exposure, and genetic backgrounds.

Study methods also vary across the included studies. The type of dietary fiber measurement varies. Some studies rely on self-reported dietary questionnaires, while others may use more objective laboratory analyses. This can lead to differences in the accuracy and precision of dietary fiber assessment. Additionally, the definition of CVD varies, which may result in inconsistent identification of cases. The duration of follow-up also varies among studies, which can influence the detection of long-term effects of dietary fiber on CVD. This heterogeneity may limit the generalizability of our findings. The diverse populations and methods make it challenging to draw broad conclusions applicable to all settings. Future research should aim to standardize study methods and target more homogeneous populations to better understand the true relationship between dietary fiber and CVD.

### 4.5. Impact of Regional Differences on Results

Regional differences in diet and culture can significantly influence the relationship between dietary fiber and CVD, as seen in the included studies. Different regions around the world have distinct dietary patterns. For example, the Mediterranean diet, prevalent in countries like Greece, Italy, and Spain, is rich in fruits, vegetables, legumes, whole grains, and olive oil. This diet provides a diverse range of fiber sources, with a high intake of both soluble and insoluble fiber. In contrast, the Western-style diet, common in many industrialized countries, is often characterized by higher consumption of processed foods, red meat, and saturated fats, and a relatively lower intake of fiber-rich foods. These differences in dietary patterns can lead to variations in the overall fiber intake and the types of fibers consumed. Populations following the Mediterranean diet may have a higher intake of dietary fiber from legumes and vegetables, which have been associated with beneficial effects on CVD. The Western-style diet, on the other hand, may provide less fiber, potentially diminishing the protective effect of fiber on CVD.

Cultural factors also play a role. In some Asian cultures, traditional food-preparing methods, such as fermenting soybeans to make products like tempeh and miso, can alter the fiber structure and bio-availability. Fermentation may increase the solubility of certain fibers, making them more easily digestible and potentially enhancing their influence on CVD. In contrast, in some Western cultures, food-processing techniques may strip away fiber from grains during milling, reducing the fiber content in the final products. Additionally, cultural eating habits, such as the frequency of meals and the consumption of snacks, can affect the overall intake of fiber-rich foods. These cultural differences can contribute to variations in the observed effects of dietary fiber on CVD across different regions.

### 4.6. Differential Effects of Fiber Sources

The differential effects of various fiber sources on CVD, as observed in the studies, warrant further exploration. The composition of fiber types within different sources may explain the varying effects. Grains, for example, are often rich in insoluble fiber, such as cellulose. Insoluble fiber has important functions in promoting bowel regularity, but its direct impact on CVD risk factors may be different from that of soluble fiber. Legumes, on the other hand, contain a significant amount of soluble fiber, such as pectin and gums. Soluble fiber can form a viscous gel in the gut, which may help lower cholesterol levels by binding to cholesterol-containing substances and preventing their absorption. This difference in fiber composition could potentially explain why legumes show more significant effects on CVD compared with grains.

The presence of other bioactive compounds in these fiber-rich foods can also interact with the fiber and influence the overall impact on CVD. Nuts are not only a source of dietary fiber but also contain high levels of healthy fats, such as monounsaturated and polyunsaturated fats. These fats may have their own independent effects on CVD risk factors, such as reducing inflammation and improving lipid profiles. However, this complexity can confound the relationship between the fiber in nuts and CVD. In contrast, vegetables contain a wide range of vitamins, minerals, and antioxidants in addition to fiber. These additional compounds may work synergistically with fiber to exert beneficial effects on CVD. For example, the antioxidants in vegetables can help reduce oxidative stress, which is a contributing factor in CVD development. This interaction between fiber and other bioactive compounds may explain why some fiber sources, such as nuts, do not show as significant effects on CVD as legumes and vegetables.

### 4.7. Limitations

In the study [21], the participants’ dietary fiber intake data were collected by researchers by using a food frequency questionnaire (FFQ). They categorized the participants into four quartiles based on their dietary fiber intake and monitored their health status for an average duration of six and a half years. The study accounted for various confounding factors, including age, gender, smoking status, physical activity level, and energy intake. The dietary fiber intake in the studies [22,23,25,26] was evaluated through an FFQ, and the occurrence of cardiovascular events among the participants was monitored for approximately five years. The study provides further evidence supporting the favorable impact of adhering to a Mediterranean diet pattern, particularly emphasizing the consumption of high-fiber foods, on cardiovascular health. Nevertheless, it is important to address potential reporting bias associated with the FFQ. The reliance on self-reports from participants in the studies [24,27] may introduce potential reporting bias.

There are limitations in the studies. Firstly, the research encompasses diverse regions worldwide, covering Asia, Europe, and North America. The populations living in these regions have distinct dietary habits influenced by race and religion, resulting in variations in the types of dietary fiber consumed in varied geographical environments. Secondly, individuals’ daily intake of dietary fiber comes from multiple sources, such as fruits, vegetables, legumes, and grains rather than a single source. The intricate interactions between different types of dietary fiber remain incompletely understood. Lastly, a balanced and healthy diet should prioritize diversity without excessively emphasizing any specific nutrient component. Additionally, it is crucial to consider other lifestyle factors for subjects with CVD, such as smoking, alcohol consumption, sedentary lifestyle, physical exercise, and psychological stress.

### 4.8. Future Studies

There is a pressing need for well-designed experimental studies to confirm the mechanisms through which dietary fiber impacts cardiovascular disease (CVD). While our review has identified potential associations, the exact physiological pathways still remain to be fully elucidated. For example, experimental models, such as animal studies or in vitro cell-based experiments, could be utilized to isolate the effects of different types of dietary fiber. These studies could control the intake of specific fiber sources and closely monitor the changes in CVD-related biomarkers, such as lipid profiles, blood pressure, and markers of inflammation. By controlling the experimental conditions, researchers can better understand how dietary fiber interacts with the body’s metabolic and immune systems to influence CVD risk.

Given the influence of regional and cultural differences on the relationship between dietary fiber and CVD, future research should delve deeper into these aspects. Cross-cultural studies could be designed to explore how different dietary and cultural practices alter the impact of dietary fiber on CVD. This could involve comparing populations with distinct dietary patterns, such as those in Asia, Africa, and the Americas, to understand how cultural factors like food preparation, eating habits, and traditional beliefs interact with dietary fiber intake and affect CVD risk.

With the increasing interest in sustainable food sources, there is a potential to explore novel fiber sources, such as those from insects or seaweeds. Research could investigate the nutritional properties and health effects of these novel fibers, especially their relation to CVD. Additionally, studying the effects of combinations of different fiber sources may provide insights into potential synergistic effects that could offer enhanced protection against CVD.

## 5. Conclusions

Within this framework, dietary fiber emerges as an extremely promising determinant of well-being. Increasing the intake of dietary fiber could help reduce the risk of CVD. Attention should be paid to consuming soluble dietary fiber found in legumes, vegetables, and fruits.

## Figures and Tables

**Table 1:** Characteristics of the included studies (*n = 7*).

References	Launch Year	Disease Types	Country/ Region	Age of Subjects	Sample Size	Observation Period
Mirmiran, P. et al. [21]	2016	CHD	Iran	38.2 ± 13.4	2295	2 years
Buil-Cosiales, P. et al. [22]	2016	CVD	America	55–80	7216	5 years
Zazpe, I. et al. [23]	2016	CVD	Spain	38.8	17,424	10 years
Partula, V. et al. [24]	2020	CVD	France	≥18	107,337	2 years
Janzi, S. et al. [25]	2020	Aortic Stenosis	Malmö	57–58	26,063	20 years
Yang, J. et al. [26]	2022	CHD	China	51.5	461,047	4 years
Kelly, R. et al. [27]	2023	CVD	England	37–73	110,497	9 years
